# Resting-state functional near-infrared spectroscopy in neurodegenerative diseases – A systematic review

**DOI:** 10.1016/j.nicl.2025.103733

**Published:** 2025-01-17

**Authors:** Franziska Albrecht, Alexander Kvist, Erika Franzén

**Affiliations:** a Division of Physiotherapy, Department of Neurobiology, Care Sciences and Society, Karolinska Institutet, Stockholm, Sweden; b Karolinska University Hospital, Women’s Health and Allied Health Professionals Theme, Medical unit Occupational Therapy & Physiotherapy, Stockholm Sweden; c Stockholm’s Sjukhem Foundation, Stockholm, Sweden

**Keywords:** Systematic review, Resting-state, Functional near-infrared spectroscopy, fNIRS, Neurodegenerative disease, Functional connectivity, Mild cognitive impairment

## Abstract

**Objective:**

To systematically review and summarize alterations found in resting-state activity as measured via functional near-infrared spectroscopy (fNIRS) in neurodegenerative diseases.

**Background:**

fNIRS is a novel and emerging neuroimaging method suitable for a variety of study designs. Resting-state is the measure of brain activity in the absence of a task, which has been investigated for yielding information about neurodegenerative diseases, mainly using magnetic resonance imaging. We aimed to systematically review the usage of resting-state fNIRS (rsfNIRS) in neurodegenerative diseases.

**Inclusion criteria:**

Studies investigating people diagnosed with a neurodegenerative disease and resting-state activity obtained with fNIRS using at least two channels.

**Methods:**

We searched three databases for publications. After the screening, 16 studies were included in the systematic review. The quality of the studies was assessed, and data were extracted. Data were qualitatively synthesized and in the case of at least 10 similar studies, a meta-analysis was planned.

**Results:**

Most studies investigated Mild cognitive impairment (50%), followed by Alzheimer’s disease (25%). Other neurodegenerative diseases encompassed Parkinson’s disease, Multiple sclerosis, and Amyotrophic lateral sclerosis. All studies reported oxygenated hemoglobin. Still, studies were heterogeneous in terms of study design, measurement duration, fNIRS device, montage, pre-processing, and analyses. A meta-analysis was not considered possible due to this heterogeneity.

**Conclusion:**

rsfNIRS shows promise in neurodegenerative disease, as most studies have observed resting-state alterations when compared to healthy controls. However, inconsistencies across studies limit data comparison and meta-analysis. Hence, we strongly advocate the application of fNIRS reporting guidelines and the establishment of rsfNIRS-specific guidelines. This will ensure reliable and comparable results in future research.

## Highlights

• A systematic review on resting-state fNIRS in neurodegenerative diseases.
• Sixteen studies were included and 50% investigated Mild cognitive impairment.
• All studies reported oxygenated hemoglobin but were otherwise heterogeneous.
• Resting-state fNIRS shows promise in neurodegenerative disease.
• Still, we strongly advocate the application of fNIRS reporting guidelines.

## 1 Introduction

### 1.1 Functional near-infrared spectroscopy for neuroimaging

Functional near-infrared spectroscopy (fNIRS), as a neuroimaging technique to measure brain function, is recently gaining more attention. Yet, functional magnetic resonance imaging (fMRI) is seen as the gold standard for functional neuroimaging, even though measurement takes place in a not ecologically valid environment, i.e., lying down in an isolated, loud tunnel. The fNIRS system can be used wirelessly and has several advantages: flexible and easy to use in an open-air environment, able to measure cortical activity at a spatial resolution of 2–3 cm, and in general no contraindications (Pinti et al., 2020). This is why it gained popularity and is usually applied to task-related or movement paradigms (e.g.; Marusic et al., 2019, Kvist et al., 2023). This popularity also led to the need to develop best practice guidelines for describing methods of fNIRS studies, which have been recently established by the Society for Functional Near-Infrared Spectroscopy (Yücel et al., 2021). An fNIRS system consists of a cap with several light sources and detectors. The fNIRS sources emit light continuously at near-infrared frequencies and the brain blood hemoglobin absorbs light of different wavelengths. The detectors then capture the reflected light. Thus, by using at least two different wavelengths, fNIRS captures brain activity by measuring changes in the concentration of oxyhemoglobin (HbO_2_) and deoxyhemoglobin (HbR) in the brain blood which can be calculated using the modified Beer-Lambert law (Cope and Delpy 1988).

### 1.2 The potential of functional near-infrared spectroscopy to measure resting-state

Resting-state activity in fNIRS and fMRI is based on the measure of Blood-Oxygen-Level-Dependent signal, i.e., BOLD. Biswal et al. (1995) were the first to obtain low-frequency fluctuations in fMRI signals from resting human participants that showed spatial correlation patterns of a neurophysiological origin. This so-called resting-state can be used to investigate the functional integration of local or whole-brain areas by measuring inter- and intra-regional temporal synchronization. Mostly, the resting-state is assessed as functional connectivity which is the “temporal synchronization of spontaneous neuronal activity in separated anatomical areas” (Sporns 2013). Further methods to analyze resting-state activity have been applied to fMRI as well as fNIRS such as graph theory. It has been shown that graph theory brain networks from rsfNIRS are stable and comparable to networks obtained by other neuroimaging methods (Niu et al., 2012). Moreover, in a recent review, it was concluded that fNIRS is a valid tool for obtaining resting-state activity in healthy as well as several diseases (Niu and He 2014).

### 1.3 Resting-state in neurodegenerative diseases

The potential of low spontaneous fluctuation – resting-state – as a diagnostic biomarker for neurodegenerative diseases has been explored for many years now (Zhou et al., 2017, Hohenfeld et al., 2018, Filippi et al., 2019). Attempts to establish disease-specific alterations have had limited success, one reason being MRI scanner differences (Teipel et al., 2017). Nevertheless, some group studies and meta-analyses showed some promising results regarding disease assessment and diagnosis using resting-state of people with Mild cognitive impairment, Alzheimer’s disease, and Parkinson’s disease that could be further developed for diagnostic biomarkers (Li et al., 2015, Gu et al., 2022). A review of dynamic functional connectivity to analyze resting-state highlighted the potential to understand the pathophysiology of neurodegenerative diseases (Filippi et al., 2019). This included predicting conversion to Alzheimer's disease from mild cognitive impairment and predicting disease severity in Parkinson's disease. Still, the review also showed the dependence on specific data processing and methodological aspects. A general assessment of dementias and their resting-state network differences to healthy aging have been summarized recently, showing severe disruptions in nearly all networks in dementia syndromes (Ranasinghe and Mapa 2024). Nevertheless, a clinically applicable rsfMRI biomarker is still far from being in sight since MRI is not eligible for all patients, scanner differences are influential as well and substantial processing of the data is needed. FNIRS has a higher potential to be implemented in clinical and diagnostic routines since it is easier to obtain and less costly than MRI. Still, it is a long way to clinical implementation. To pave this way, it is of high importance to summarize the research that has been done so far to understand the research evidence, gaps, and quality of rsfNIRS in neurodegenerative diseases. While two systematic reviews have summarized task-based and rsfNIRS studies in Mild cognitive impairment and dementias including Alzheimer’s disease (Yeung and Chan, 2020, Wang et al., 2024), and another reviewed rsfNIRS in general (Niu et al., 2013), no systematic reviews or meta-analyses on other neurodegenerative diseases exist.

### 1.4 Aim and hypothesis

We aimed to identify, summarize, and systematically analyze the evidence on rsfNIRS in neurodegenerative diseases to review the current application, methods, and findings. If at least ten studies show considerable homogeneity, changes in connectivity metrics compared to healthy controls will be meta-analyzed. Otherwise, data will be qualitatively synthesized. We aimed to answer the questions of which neurodegenerative conditions have been investigated, which fNIRS systems and channel locations have been used, how was the data analyzed, and what are the main findings.

## 2 Materials and methods

### 2.1 Study selection

#### 2.1.1 Search and article selection

**Fig. 1:**
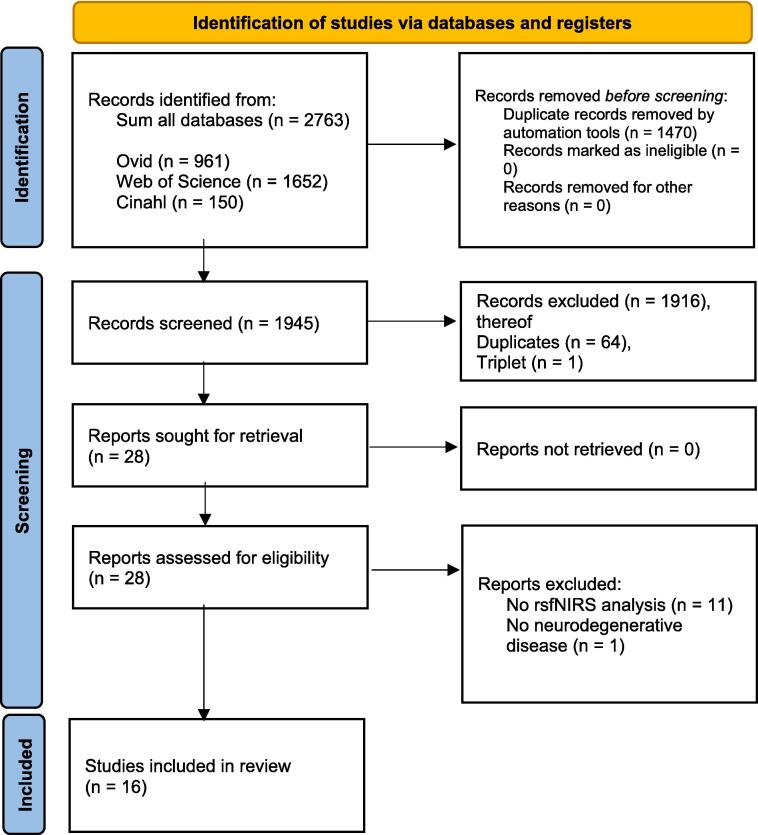
PRISMA statement flow diagram. The flow of information through different phases of the systematic literature search identifying rsfNIRS in neurodegenerative diseases. The image was modified according to the PRISMA statement (Moher et al., 2009).

To assure high quality and reproducibility, literature search and study selection were performed according to the Preferred Reporting Items for Systematic Reviews and Meta-Analyses (PRISMA) statement (https://www.prisma-statement.org) (Fig. 1). Scientific librarians at the Karolinska Institutet University Library performed the literature search in the following databases: Medline, Web of Science, and Cinahl. After the original search was performed on 5 August 2020, the search was last updated on 31 May 2021 according to published methods (Bramer and Bain 2017). The last search was conducted on 15 November 2022. The search strategy was developed in Medline (Ovid) in collaboration with the librarians. For each search concept Medical Subject Headings (MeSH-terms) and free text terms were identified. The search was then translated, in part using Polyglot Search Translator, into the other databases (Clark et al., 2020). The strategies were peer-reviewed by another librarian before execution. De-duplication was done according to published methods (Bramer et al., 2016). One final, extra step was added to compare DOIs. Additionally, a recent systematic review on rsfNIRS in Mild Cognitive Impair was checked to include references and citations of eligible studies, but no further study was added (Yeung and Chan 2020). The full search strategies for all databases are available in the Supplement. Study selection was conducted by two authors (FA and AK) based on titles and abstracts using Rayyan (Ouzzani et al., 2016.

#### 2.1.2 Inclusion and exclusion criteria

Research of any type of setting was considered for the systematic review. We did not constrain our search regarding study design, nor was an intervention required. If an intervention study was included, we only regarded the baseline measurement of rsfNIRS. Only peer-reviewed, English-language, original, full-length journal articles were included. Studies needed to include measurement of cortical activity utilizing fNIRS during rest (standing, sitting, or lying down without a motor or cognitive task), in any cortical region with at least two measurement channels. Studies on human participants with any type of neurodegenerative disease were considered. We disregarded studies with participants under 18 years of age.

### 2.2 Data extraction main outcome, and analysis

The main outcome was any rsfNIRS activity measure in people with a neurodegenerative disease, including most common ones such as effective connectivity (dynamic causal modeling, structural equation modeling), functional connectivity (correlation, mutual information, cross-correlation, granger causality, transfer entropy, coherence, phase locking, phase consistency, phase slope), or graph theory measures (Table 1).

**Table 1:** Demographics, clinical values, and study design of the included studies.

(See above-mentioned references for further information.)

Further, we extracted general data about the authors, year of publication, sample size, demographics, and neurodegenerative disease type. Extracted data about the methodology were: fNIRS system and the optode number, optode locations, scanning protocol (time, setting, position, instructions), reported signal quality metrics and rejection criteria, pre-processing (filtering, denoising), analysis method, and statistical thresholding. If available, extracted connectivity/activity data for the *meta*-analysis included measure value, effect size, and corresponding reported location (e.g., seed region or channel pair).

### 2.3 Risk of bias and quality assessment

Quality assessment was carried out using the JBI Critical Appraisal Checklist for Analytical Cross-Sectional Studies with eight different criteria (Moola et al., 2017), which has been used in similar reviews (Li et al., 2022, Ulugut et al., 2023). Two authors (FA and AK) conducted quality assessments independently, with disagreements on the quality score being settled by discussion (Table 2).

**Table 2:** Quality assessment of the studies. Adapted from JBI Critical Appraisal Checklist for Analytical Cross-Sectional Studies.

### 2.4 Meta-analysis

We planned to perform a meta-analysis in case of a sufficient recommended number of similar studies, i.e., ten studies (Müller et al., 2018). For a study to be included in the meta-analysis, it must report optode locations according to a common reference system (10–20 EEG, MNI, Broadman Areas, or similar). Only studies contrasting neurodegenerative diseases to healthy controls were regarded.

### 2.5 Data analysis

**Fig. 2:**
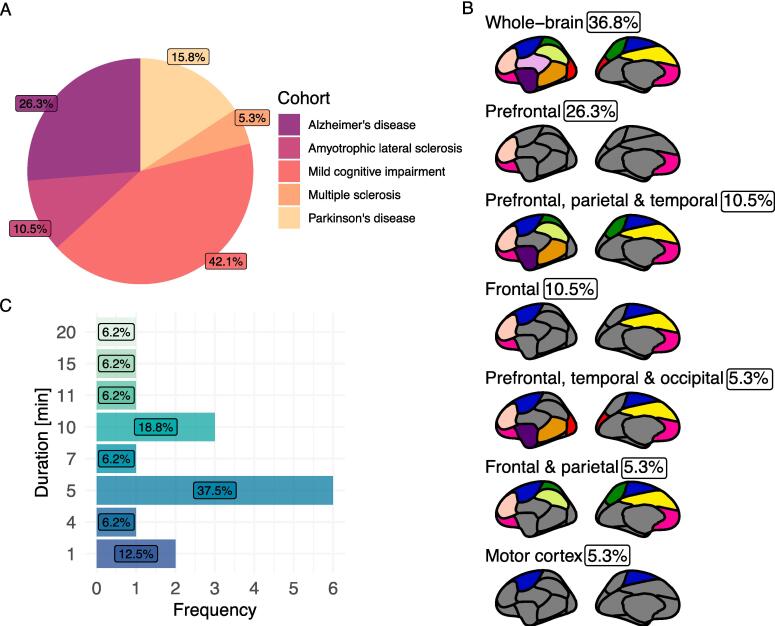
Summarizing the main outcomes of the systematic review. **A:** Pie chart showing the percentage distribution of neurodegenerative disease cohorts. **B:** Frequency of measurement duration (minutes) applied in the rsfNIRS studies. **C:** Distribution of brain areas covered in percentage of the included rsfNIRS studies. Approximation overlaid on the ggseg Chen brain template.

To create Fig. 2, data was imported to R. The pie chart (A) was built using ggplot2, and percentages of researched disease groups out of the 16 included studies were calculated (Wickham 2016). Note that some studies included more than one disease which is why we refer to cohorts instead of studies. The 16 studies comprised 19 cohorts. For the brain plot (B), we extracted the covered brain regions from the respective studies. Through ggseg in R, we used the Chen atlas as a template to visualize the approximated regions (Mowinckel and Vidal-Piñeiro 2020). The bar plot (C) was built in ggplot2 by calculating the frequencies and percentages for the measurement duration in minutes.

### 2.6 Data availability statement

The protocol was registered at PROSPERO (https://www.crd.york.ac.uk/prospero/display_record.php?ID=CRD42022364752). All included studies are available through the respective article databases, no additional data were requested from the authors of the original studies. The scripts and data used for the Fig. 2 building can be found on the OSF (https://osf.io/5yfr4/).

## 3 Results

### 3.1 Search and quality

The search identified 2763 studies, whereof we screened 1945 and finally included 16 studies (Fig. 1, Table 1). There were not enough homogenous studies to meta-analyze the results. The quality assessment did not lead to the exclusion of a study, even though the quality was varying across studies (Table 2).

### 3.2 Demographics

The included studies originated from seven countries. Five studies were done in China, followed by four in South Korea, two in the USA, and two in Germany. Three research groups published multiple studies on different participant cohorts that were included in this review, totaling six studies (Li et al., 2018, Ghafoor et al., 2019, Nguyen et al., 2019, Niu et al., 2019, Yang and Hong, 2021, Ho, Kim et al. 2022). The remainder of the studies were produced by unique research groups. All studies were published between 2014 and 2023. The distribution of the publication years was skewed towards 2019 and 2022, where four studies were published per year. In 2014 and 2018, two studies were published per year, whereas in the years 2016, 2020, 2021, and 2023 only one per year was published.

Most investigated was Mild cognitive impairment, while Multiple sclerosis was only investigated once (Fig. 2A). Two studies investigated more than one neurodegenerative disease, which is why we refer to cohorts instead of studies in Fig. 2A. The studies included a median of 23 participants with neurodegenerative disease, but the included patients' number range was broad (N = 3–64). The median age was 69.27 years, ranging between 53.70–75.90 years. Thirteen studies compared their results to healthy controls. A median of 29 healthy controls was included in those studies, ranging from 8 to 64 healthy controls per study. Two studies had further a within-disease subtype comparison, two studies additionally involved another neurodegenerative disease as a comparison, and one did not compare brain networks to a reference group. The included healthy control groups were matched in terms of sex, age, and education in one study. Two studies matched their healthy controls concerning age and sex, while four studies only reported age matching. Five studies did not mention a procedure for ensuring a well-matched healthy control group, and two compared within disease without mentioning a matching process.

### 3.3 Main outcome

Connectivity measures were reported in nine studies, whereof the majority reported functional connectivity (Pearson correlations between all channels and seed-based, N = 8) and graph theory (N = 2) (Table 1). Another connectivity measure was effective connectivity (N = 1). Seven studies reported different kinds of hemoglobin activity or spectral power measures such as changes in HbO_2_ over measurement duration or amplitude of low-frequency fluctuations.

### 3.4 Technical and study design

All included studies reported HbO_2_. The majority reported additionally HbR (N = 10) and only four studies reported total hemoglobin (HbT). None reported any other hemoglobin measures such as hemoglobin difference or the correlation-based signal improvement (CBSI) measure (Cui et al., 2010). Montages were mainly standardized according to the 10–20 EEG standard (N = 11). The number of channels differed along with technical advancement during the years the studies were conducted as well as the purpose of the studies (i.e., technical investigation or group-wise differences) (range channels = 2–188). Along the same line, the number of channels determined the brain areas that could be covered. Studies comprised the whole brain (N = 5) and region-of-interests such as frontal (N = 3) or only prefrontal parts (N = 6) (Fig. 2B). The remaining studies collected parietal or temporal areas. One study collected the whole brain and split it into two parts, i.e., the frontal and parietal areas, separately. There was a broad variety of fNIRS devices, models, and manufacturers, with no more than two studies using the same type of device.

Study designs were generally very heterogeneous. Three studies did not report about the resting-state study design such as a lack of setting description or description of eyes closed or opened. Nine studies reported eyes closed, two reported using a fixation cross, and five did not report their setting. Darkened or dimly lit rooms were reported in five studies, and one study had ambient background noise during measurement. Five studies reported explicitly that their participants were lying (N = 2) or sitting (N = 3) during measurement, while the remainder did not report positioning. Measurement duration spanned between 1 and 20 min, whereas the majority collected 4–5 min of data (N = 7) followed by 10–11 min (N = 4) (Fig. 2C). Noteworthy is that two studies measured only 1 min.

### 3.5 Settings and processing

The wavelengths employed were always two, except for one study that used three. The respective study used a combination of 780, 808, and 850 nm (Bu et al. 2019). Further, the used wavelengths varied slightly for HbO_2_, where seven different wavelengths were used. The majority employed 695 nm (N = 4), followed by 780 and 730 (N = 3), 690 and 760 (N = 2), and 750 and 670 (N = 1). For HbR, eight studies applied 850 nm and seven 830 nm.

The sampling rate was mainly around 10 Hz (7.81–10 Hz, N = 11). Two studies did not report the sampling rate. Interestingly, two studies used frequency-modulated systems, one reporting a demodulated sampling rate of 50 Hz, and the other one not reporting the demodulated sampling rate. Ten studies reported signal rejection criteria, three reported how many channels or participants were excluded after rejection, and only one study reported signal quality metrics of the dataset.

General preprocessing steps of the data were reported in all studies but not transparently regarding the details of how and which software was used in many studies. Eight studies transparently reported used software toolboxes and processing parameters, of which only one reported in detail which software functions were used for preprocessing. Within the sixteen studies, there was a wide variety of applied processing software, with no two studies using the same processing pipeline or toolbox. Four studies did not report the software, while four used custom scripts. Regarding the significance of the results, six studies corrected for multiple comparisons (Bonferroni, false discovery rate, or family-wise error), one thresholded the t-maps at 50 %, seven had no correction of alpha level, and one did not report the threshold.

### 3.6 Resting-state alterations in neurodegenerative diseases

In Mild cognitive impairment, the eight cohorts had no converging results due to the investigation of different rsfNIRS metrics and regions of interest (Table 3). Seven found decreased resting-state connectivity or activity, comprising the prefrontal, parietal, occipital, and temporal cortices. Contrarily, two studies found increased prefrontal connectivity or signal variability strength in Mild cognitive impairment when compared to healthy controls (Nguyen et al., 2019, Niu et al., 2019).

**Table 3:** Summary of the rsfNIRS alterations found in the included studies.

Author year	fNIRS metric	Results direction and significance	Results explanation
**MCI**			
Bu et al. 2019	Effective connectivity	MCI < HC p < 0.05, uncorrected	MCI: lower coupling strength between brain regions associated with prefrontal lobes than in HC
Ghafoor et al. 2019	Functional connectivity & graph theory	MCI < HC No significance testing	MCI: lower mean prefrontal global network efficiency (28 %) than HC (54 %)
			Lower prefrontal nodal clustering coefficient and degree centrality in MCI than HC
Nguyen et al. 2019	Functional connectivity	MCI > HC p < 0.05, Bonferroni	MCI: higher right and inter-hemispheric prefrontal connectivity
Yang et al. 2021	Functional connectivity & graph theory	MCI < HC No significance testing	MCI: weaker prefrontal functional connectivity than HC
**Zeller et al. 2018**	Spontaneous low frequency oscillations	MCI < HC elderly, HC elderly < HC young	MCI and elderly HC: less spontaneous low frequency oscillations in the frontal cortex than young controls
			MCI: decreased low frequency oscillations in parietal cortex compared to elderly HC
			Young and elderly HC: less spontaneous low frequency oscillations in the frontal cortex in elderly controls
Zhang et al. 2022	Functional connectivity	MCI < HC p < 0.05, FDR	MCI: significantly reduced connectivity between bilateral prefrontal, parietal, occipital, and right temporal lobes than in HC
**MCI & AD**			
Niu et al. 2019	Functional connectivity (dynamic & static)	MCI = AD = HC, MCI & AD > HC p < 0.005, uncorrected	No differences in static functional connectivity between all groups
			AD: dynamic functional connectivity variability strength higher than in HC
			MCI: dynamic functional connectivity variability strength higher than in HC. No differences to AD
Li et al. 2018	Multiscale entropy	AD < HC, AD < MCI, MCI = HC p < 0.05, Bonferroni	AD: Reduced signal complexity in default mode, frontoparietal, ventral, and dorsal attention networks compared toHC
			MCI: no difference when compared to HC
			AD and MCI: Lower signal complexity in default mode network in AD than in MCI
**AD**			
**Ferdinando et al. 2023**	Spectral entropy	AD > HC p < 0.05, uncorrected	AD: higher spectral entropy in bilateral prefrontal cortices than in HC
Ho et al. 2022	Mean HbO_2_	AD amyloid = AD prodromal = ADD p < 0.05, uncorrected	No significant differences in prefrontal HbO_2_ concentrations
Keles et al. 2022	Standard deviation of HbO_2_ changes	AD < HC No significance testing	AD: lower hemodynamic activity in bilateral prefrontal cortices
**PD**			
Eggebrecht et al. 2014	Seed-based correlation	PD, no comparison to other cohorts/ no significance testing	No comparison to HC, but in PD: sensory, auditory, and motor subnetworks displayed robust inter-hemispheric functional connectivity with contralateral homotopic regions.
Wang et al. 2022	Amplitude of low frequency fluctuations	PD drug < PD without p < 0.05, FDR	PD drug: lower amplitude of low frequency fluctuations in dorsolateral prefrontal cortex, ventrolateral prefrontal cortex and orbitofrontal cortex than in PD without.
**ALS**			
Deligani et al. 2020	Functional connectivity	ALS > HC p < 0.05, FWE	ALS: higher interhemispheric and right intra-hemispheric connectivity in the frontal and prefrontal cortices than in HC
Kopitzki et al. 2016	Functional connectivity	ALS ≠ HC p < 0.05, Bonferroni	ALS: no significant differences in homotopic functional connectivity maps to HC. Correlations between frontal and temporo-occipital areas, and anterior temporal lobes with orbitofrontal and posterior-temporal areas were different to HC.
			HC: homotopic functional connectivity between occipital and posterior temporal cortices
**MS**			
Jimenez et al. 2014	Total hemoglobin coherence	MS = HC p < 0.01, uncorrected	MS: no significant differences in resting-state coherence in motor cortex in comparison to HC

Explanation of signs: ≠ alteration but no direction reported, = no difference, < decrease, > increase. Abbreviations: AD = Alzheimer’s disease, ADD = AD with deterioration of memory, language, social abilities, AD amyloid = cognitively normal, but amyloid positive PET, AD prodromal = AD with mild brain dysfunction, ALS = Amyotrophic lateral sclerosis, FDR = False discovery rate, FWE = family-wise error, HbO_2_  = oxygenated hemoglobin, HC = healthy controls, MCI = Mild cognitive impairment, MS = Multiple sclerosis, PD = Parkinson’s disease, PD drug = PD tremor-dominant with trihexyphenidyl, PD without = idiopathic PD without trihexyphenidyl.

Alzheimer’s disease was investigated in five studies, where one study compared different stages of Alzheimer’s disease (Table 3). Noteworthy, the respective study did not find differences in rsfNIRS between the stages of Alzheimer’s disease (Ho et al., 2022). The results for the remaining studies were not homogeneous, i.e., both increases and decreases in resting-state were found in different brain areas. One study found reduced signal complexity in several networks (Li et al., 2018) and another obtained lower activity in the prefrontal cortex compared to healthy controls (Keles et al., 2022). On the contrary, higher prefrontal activity (Ferdinando et al., 2022) and stronger dynamic connectivity variability in and between various networks have been found compared to healthy controls (Niu et al., 2019).

The two studies of Parkinson’s disease (Table 3) did not compare findings with healthy controls. One study aimed at obtaining robust and common networks in Parkinson’s disease using rsfNIRS (Eggebrecht, Ferradal et al. 2014) but did not include any reference group. Another study compared tremor-dominant people with Parkinson’s disease who were medicated with a specific drug to those who were non-medicated. The resting-state activity in the prefrontal cortex was weaker in medicated than in those who were not medicated (Wang et al., 2022).

The two studies in Amyotrophic lateral sclerosis found alterations in functional connectivity when compared with healthy controls (Table 3). One study identified increased connectivity in and between frontal and prefrontal regions compared to healthy controls (Deligani, Hosni et al. 2020). The second study found no differences in homotopic resting-state functional connectivity maps (Kopitzki, Oldag et al. 2016). Still, patterns of functional connectivity were different from healthy controls showing correlations in Amyotrophic lateral sclerosis between the frontal and temporal-occipital areas, as well as anterior temporal lobes with orbitofrontal and posterior-temporal areas. In healthy controls, a correlation between occipital and posterior temporal cortices was obtained.

There was only one study on Multiple sclerosis that could not show alterations in resting-state coherence in the motor cortex compared to healthy controls (Jimenez, Yang et al. 2014) (Table 3).

## 4 Discussion

We identified 16 studies of rsfNIRS activity in participants with neurodegenerative diseases and reviewed study designs and methods. We qualitatively synthesized the results since the study design, rsfNIRS metric, and populations were too heterogeneous to conduct a meta-analysis. For the main resting-state activity outcome, most of the studies reported functional connectivity, but a plethora of analysis methods were used. All studies reported at least HbO_2_. The majority of studies covered the whole brain, but many focused on region-of-interests. Mild cognitive impairment and Alzheimer’s disease were the most investigated neurodegenerative diseases. Nearly all included studies found alterations in neurodegenerative diseases compared with healthy controls. Only one study investigating eight participants with Multiple sclerosis did not find differences in motor cortex resting-state activity when compared with healthy controls.

### 4.1 Resting-state alterations in neurodegenerative diseases

Noteworthy, all included studies reported HbO_2_ and the majority used a common reference system like the 10–20 EEG. Further, nearly all studies found resting-state alterations, both increases and decreases, compared with healthy controls. Thus, rsfNIRS seems to capture resting-state alterations related to neurodegenerative diseases. In Mild cognitive impairment, studies mainly found resting-state connectivity reductions in frontal regions. Hence, Mild cognitive impairment seems to be the only neurodegenerative disease that shows evidence of consistent findings across different studies. Both fNIRS and fMRI use the Blood-Oxygen-Level-Dependent signal, i.e., BOLD as a proxy to measure neuronal activity. A study in Alzheimer’s disease showed that reduced MRI BOLD-based functional connectivity might not only be based on neuronal alterations but also on vascular-hemodynamic processes as measured by pulsed arterial spin labeling (Göttler et al., 2019). The authors also suggest that a decrease in cerebral blood flow and oxygenation gives rise to decreased BOLD. This relationship might only be true in Alzheimer’s disease and not in healthy controls. In another study about the basis of BOLD, a proxy measure of delayed cerebral blood flow, capillary transit time heterogeneity, was negatively related to BOLD (Schneider et al., 2022). This relationship has been shown in asymptomatic patients with unilateral high-grade internal carotid artery stenosis. For the remaining neurodegenerative diseases in our systematic review, alterations that were found to be consistent across studies of the same disease were hard to find. All studies found some kind of alteration, increase or decrease, except for one. However, anatomical overlaps within the disease cohorts were limited due to heterogeneity in montages/ brain areas covered and analysis methods. This shows that so far, there is no consistent evidence of findings. Thus, the implementation of rsfNIRS in the clinical assessment or even as a biomarker seems not to be in the near future.

### 4.2 Study designs and adherence to best practice guideline

Efforts have been made to ensure good scientific practice in fNIRS research. Yücel et al. (2021) published very thorough recommendations including a checklist on how to report fNIRS studies. Important to mention is that only five of the studies included in the present review were published after 2021. Some specific resting-state recommendations are mentioned but the incorporation of Yücels guidelines is only moderate among those post-2021 studies. These studies fulfill the criteria concerning technical reporting, e.g., hemoglobin measure, duration, and wavelength. However, the study design was only described sufficiently in one study (Wang et al., 2022), even though this one omitted to report the sampling rate. Regarding study design, it is recommended to mention the recording duration, participants’ surroundings comprising lights, acoustics, eyes closed or open with a fixation point, and the participants’ instructions. Especially the instruction on how the authors defined “resting-state” is not reported in the majority of the studies. Another important point is that studies varied between eyes closed, eyes open, or eyes-on-a-fixation-cross instruction. In rsfMRI, it has been shown that brain network rest-state activity significantly differs between those conditions (Agcaoglu et al., 2019). During the eyes-on-fixation-cross condition, there was an increased connectivity within visual networks and to other networks. During the eyes-closed condition, increased connectivity was found within the auditory and sensorimotor networks and their connectivity to other networks. In the present review, one study, which did not report on the eye condition, found decreases in the occipital lobe in Mild cognitive impairment compared with healthy controls (Zhang et al., 2022). These differences and lack of description of instructions lead to significantly different results between studies and make them incompatible to draw conclusions.

Another shortcoming is the documentation of the description of the optode array design, cap, and targeted brain regions, which should be reported in detail (Yücel et al., 2021). Remarkably, most studies reported that the 10–20 EEG system was used, while only two studies did not mention the basis for optode placement. Still, the exact position of the optode, e.g., Source 6 at FC2, was mentioned in 13 out of 16 studies. The lack of standardized anatomical referencing leads to anatomical inaccuracy between participants and most importantly between studies. Further, no study co-registered with MRI for exact anatomical location. The reason might be that it would increase costs, and many participants may probably be not eligible for MRI. Along the line, even though 36 % of the reviewed studies included the whole brain, whole-brain coverage is not the standard. The underlying reasons are manifold including that for covering the whole brain, many channels are necessary. One study had a workaround for this and measured the two hemispheres subsequently. This increases testing duration but should be worth considering the gain of information. There might be also good reasons to choose specific regions-of-interest for example in cases of diseases that primarily target specific brain areas. Still, whole-brain coverage would be ideal since this enables *meta*-analyses and findings in unexpected areas, i.e., besides the pre-specified regions. It could be a vicious circle if the selection of regions-of-interest is based on the results of low-sample size studies. Regions-of-interest are very specific, and there is an uncertainty in the cranio-cerebral correspondence due to the nature that usually a pre-sized cap is fitted to the participant’s scalp based on an approximation to landmarks (the majority used the 10–20 system) leading to between-subject cranio-cerebral variability. Thus, fNIRS captures cortical activity from the scalp without direct anatomical information of the participant being measured. Studies showed that the uncertainty in correspondence might be an average standard deviation of 8 mm (Okamoto et al., 2004) (For a review see Tsuzuki and Dan 2014). Noteworthy, the respective study used low-resolution MRI (1 T) and smoothed the structural MRI with 8 mm. Anatomical registration methods such as photogrammetric methods could be important to be able to compare results (Jaffe-Dax et al., 2020, Erel et al., 2021).

In a study by Aarabi and Huppert (2019), functional connectivity metrics and their dependency on the data acquisition length have been investigated. Data length influences the stability of connectivity metrics; thus, conclusions and comparisons should be drawn cautiously across different studies. This includes functional connectivity (e.g., Pearson correlations) and graph theory (topological properties of weighted functional brain networks). The authors showed that stability increases with increased length. However, in their study, the investigated length was between 30 s and 4.5 min. Two out of our 16 studies had a length between 1 and 4 min. The majority had a length of either 5 or 10 min. This means that in terms of metric stability, most studies performed well and should have obtained a stable signal. The signal stability beyond 4.5 min remains yet to be investigated. Methodological research should focus on common durations like 10 min and longer to exclude the possibility of a saturation effect-like curve.

Furthermore, the best practice guidelines recommend testing the signal quality of the raw fNIRS signal in each channel and if used for channel rejection, reporting how many channels or participants are excluded from further analysis (Yücel et al., 2021). Ten of the 16 included studies reported clear rejection criteria based on signal-to-noise ratio or other signal quality metrics, but only three reported how many participants or channels were excluded. Better reporting of signal quality metrics would improve understanding of used datasets.

In general, the topic of open and reproducible science is a less regarded one among the identified studies. None of the studies was pre-registered. Also, data and code sharing, which is also mentioned by Yücel et al. (2021), would enable reproducibility. Many studies used custom scripts and widely varying analysis methods and did not supply the code. This code sharing would be the first step to overcoming the current lack of reproducibility and replication studies. So far, we have not found any replication or test-re-test studies when synthesizing the literature for this review. In the long term, a similar evolution to the fMRI field should be aimed for. More resources should be put into the development of robust and standardized toolboxes and pipelines. In the field of MRI, a standardized pre-processing pipeline has been suggested and limits the degrees of freedom in that part of the analysis pipeline (Esteban et al., 2019).

### 4.3 Comparison to recent systematic reviews and *meta*-analysis

Studies on rsfNIRS in neurodegenerative diseases are rare, thus, systematic reviews are rare. To the best of our knowledge, two systematic reviews and *meta*-analyses have been published (Yeung and Chan 2020, Wang, Wang et al. 2024). Wang et al. (2024) reviewed 13 resting-state and task-based functional connectivity studies in Mild cognitive impairment and found mainly decreases in the prefrontal, parietal, and occipital cortex. Based on their review, the authors conclude that rsfNIRS can identify differences in functional connectivity between Mild cognitive impairment and healthy controls and foresee the implementation as a biomarker. Yeung and Chan (2020) lumped together Mild cognitive impairment and dementias, e.g., Alzheimer’s disease and thus included 36 studies of resting-state and task-based functional connectivity (12 rsfNIRS). Of note, there are substantial differences between these systematic reviews and our systematic review. Most important was the difference in the definition of “resting-state”. Yeung and Chan (2020) defined rest not as resting-state of the mind, like we did, but as a resting body, meaning sitting, standing, or lying while performing a task. Further, the two reviews included also task-based fNIRS in their conclusion, while we solely focused on rsfNIRS since task-based fNIRS is even more heterogeneous. Both studies had more lenient inclusion criteria, leading to differences in included rsfNIRS studies. To name a few, e.g., including conference abstracts, counting baseline before a task as resting-state, and deciding to *meta*-analyze two studies.

### 4.4 Limitations

We acknowledge that there could be a publication bias in the way that if no differences in resting-state activity have been found, studies might not have been published. Further, we only regarded studies that were published in English. We included a wider range of resting-state metrics, i.e., quantifying activity and not only connectivity. This might be too lenient, but we argue that this also captures the evolvement of rsfNIRS over time and is still measuring resting-state. We did not introduce criteria for the length of resting-state measurement which could lead to the inclusion of unstable results since it has been found that the metric stability depends on the signal length (Aarabi and Huppert 2019).

Due to the heterogeneity between the studies and not enough studies, we could not perform a *meta*-analysis, and thus could not evaluate the results statistically.

### 4.5 Conclusion

All 16 eligible studies on resting-state in neurodegenerative diseases reported HbO_2_. The majority found alterations in resting-state activity compared with healthy controls. We identified five different neurodegenerative diseases that have been investigated with rsfNIRS, Mild cognitive impairment and Alzheimer’s disease being the most frequently investigated diseases. But the spectrum is broad, leaving room for improvement and further research including also atypical Parkinson’s diseases or frontotemporal dementias.

Almost every study applied a common reference system for optode placement like the 10–20 EEG system. However, the study quality and findings are very varying, and not enough studies have been published for a meta-analysis. Best practices in study reporting have been published but the adherence is very limited (Yücel et al., 2021). Even though rsfNIRS is a promising tool, at the current state, we are far from finding a disease-specific biomarker due to study heterogeneity concerning study design, data acquisition, preprocessing, and analyses. More studies on resting-state in neurodegenerative diseases that adhere to reporting guidelines should be done in the future to establish rsfNIRS as a robust imaging method.

## Study funding

This study is supported by grants from the Swedish Research Council (2022–00636, 2016–01965), the Swedish Parkinson Foundation as well as the Center for Innovative Medicine (CIMED) (FoUI-975387 and FoUI-973826), and the Regional Agreement on Medical Training and Clinical Research (ALF)(RS2021-0855) between Karolinska Institutet and Region Stockholm. Further funding was provided by the Augusta and Petrus Hedlunds Stiftelse and Stohnes Stiftelse.

## CRediT authorship contribution statement

**Franziska Albrecht:** Writing – original draft, Visualization, Validation, Software, Resources, Project administration, Methodology, Investigation, Funding acquisition, Formal analysis, Data curation, Conceptualization. **Alexander Kvist:** Writing – review & editing, Validation, Methodology, Investigation, Conceptualization. **Erika Franzén:** Writing – review & editing, Supervision, Resources, Project administration, Investigation, Funding acquisition, Conceptualization.

## Declaration of competing interest

The authors declare that they have no known competing financial interests or personal relationships that could have appeared to influence the work reported in this paper.

## Data Availability

This is a systematic review, all used data has been already published and is available.
